# Interplay between polygenic risk and family processes in predicting trajectories of adolescent externalizing behaviors

**DOI:** 10.3389/fpsyt.2025.1505035

**Published:** 2025-03-12

**Authors:** Jinni Su, Belal Jamil, Kit K. Elam, Angel D. Trevino, Kathryn Lemery-Chalfant, Eleanor K. Seaton, Rick A. Cruz, Kevin J. Grimm

**Affiliations:** ^1^ Department of Psychology, Arizona State University, Tempe, AZ, United States; ^2^ Department of Applied Health Science, Indiana University, Bloomington, IN, United States; ^3^ Department of Psychology, University of Illinois Urbana-Champaign, Champaign, IL, United States

**Keywords:** polygenic, gene-environment interplay, externalizing, adolescence, ABCD study

## Abstract

**Introduction:**

There is limited understanding on how polygenic scores derived from genome-wide association studies of adult and child psychopathology may uniquely predict childhood traits. The current study took a developmental approach to examine the interplay between adult-based and child-based polygenic scores with family processes in predicting trajectories of externalizing behaviors from late childhood to early adolescence among racially-ethnically diverse youth.

**Method:**

Data were drawn from the non-Hispanic White (N = 5,907), non-Hispanic Black (N = 1,694), and Hispanic youth (N = 2,117) from the adolescent brain cognitive development (ABCD) study. Parents reported on youth externalizing behaviors at baseline (T1, age 9/10), 1-year (T2, age 10/11), 2-year (T3, age 11/12), and 3-year (T4, age 12/13) follow-up assessments. Youth reported on parenting and family environment at T1 and provided saliva or blood samples for genotyping.

**Results:**

Both polygenic scores for adult externalizing and childhood aggression predicted greater likelihood of following trajectories with higher externalizing behaviors. Among non-Hispanic White youth, polygenic scores also predicted greater family conflict, which in turn predicted higher externalizing behavior trajectories.

**Discussion:**

Our findings indicated that both adult-based and child-based polygenic scores for externalizing behaviors are useful in predicting trajectories of externalizing behaviors, highlighting developmental continuity in genetic influences. Family processes, especially family conflict, play an important role in adolescent externalizing behaviors across racial-ethnic groups, suggesting the need to target family conflict in intervention efforts. Findings also highlight the importance of conducting research in diverse populations, including improving diversity in genetically informed studies.

## 1 Introduction

Externalizing behaviors include aggressive, rule-breaking, and disruptive behavioral problems (1). These behaviors are prevalent among children and adolescents, with estimates ranging from 18-33% for children and adolescents exhibiting high levels of externalizing problems (2). Externalizing behaviors are associated with a host of negative outcomes, including increased risk for subsequent criminal and violent behavior, substance use, and lower academic performance (3, 4). Externalizing behaviors are influenced by genetic predisposition, the environment (e.g., family), and the complex interplay among them (1, 5). Understanding the etiology of externalizing behaviors necessitates a developmental approach, given that externalizing behaviors change across development and that genetic and environmental influences on externalizing behaviors can also change across development (5–7). The current study used a longitudinal design to capture heterogeneity in externalizing behaviors over time by identifying distinct trajectories of externalizing behaviors from late childhood to early adolescence. Given the salient role of the family environment in child development (8), we examined two forms of gene-environment interplay, gene-environment interaction (G×E) and gene-environment correlation (rGE), between polygenic risk for externalizing behaviors and multiple family processes (i.e., family conflict, parental acceptance, parental monitoring) in predicting trajectories of externalizing behaviors among non-Hispanic White (“White”), non-Hispanic Black/African American (“Black”), and Hispanic youth.

### 1.1 Trajectories of externalizing behaviors across adolescence

Externalizing behaviors change across development, and on average decline from childhood to adolescence, yet most of this research has been conducted in White populations whereas past literature posits racial/ethnic differences in externalizing behavior trajectories (9). Moreover, there is substantial heterogeneity where youth often follow varying trajectories of externalizing behaviors over time, where some youth may be at risk for heightened trajectories of externalizing problems (10). Most adolescents follow a low-risk trajectory, characterized by stable low levels of externalizing problems from childhood to adolescence (4). Yet, a significant proportion of adolescents may fall into other trajectories such as increasing, decreasing, or persistently high levels of externalizing problems (3, 4). Understanding risk and protective factors for higher-risk externalizing trajectories is crucial for informing prevention and intervention efforts.

### 1.2 Genetic risk for externalizing behaviors

Externalizing behaviors are heritable and have a complex polygenic architecture. Higher polygenic risk scores (PRS; aggregated effects of hundreds of thousands genetic variants across the genome) for conditions like aggression, rule breaking, and alcohol use disorder (AUD) have been linked to increased externalizing problems in White, Hispanic, and Black samples, underscoring the relevance of polygenic influence on externalizing outcomes throughout development (11–17). However, there is a notable gap in research examining how PRS derived from genome-wide association studies (GWAS) of adult psychopathology may predict childhood traits, particularly among racially and ethnically diverse youth (18). Genetic influences can change across development, so GWAS based on adult samples vs. child samples may illuminate different effects. This is critical for understanding how genetic risk unfolds across development in order to inform early prevention and intervention efforts. There is some evidence suggesting that higher PRS for psychopathology among adults, such as AUD and schizophrenia, may be associated with increased externalizing behaviors in adolescence (11, 12, 19). Recent studies showed that PRS derived from a GWAS on adult externalizing based on a sample of over one million individuals of European ancestry predicted externalizing behaviors among White adolescents (20) and greater likelihood of developing any externalizing disorder among Mexican youth (21). However, few studies have examined how PRS may predict distinct trajectories of externalizing problems in adolescence. One study found that PRS derived from GWAS on aggression among White children distinguished between stable low aggressive behavior and moderate and high-decreasing trajectories of aggressive behaviors based on parent report from latent childhood to early adulthood (22). To our knowledge, no research has examined adult-based and child-based PRS simultaneously, across racially and ethnically diverse samples, to evaluate their unique effects in predicting adolescent externalizing behaviors (23). Recent literature has highlighted this gap and called for more research in this area (7).

### 1.3 Gene-environment interplay: the role of family processes

Family is one of the most salient proximal contexts that influence child development (24). Research has linked multiple aspects of family processes to adolescent externalizing behaviors. In particular, greater family conflict has been associated with higher externalizing problems in adolescence across Black, Hispanic, and White youth (25), whereas higher levels of parental acceptance and parental monitoring are associated with lower externalizing problems (26–28).

Family processes may also influence adolescent externalizing behaviors through complex interplay with genetic factors, including gene-environment interactions (G×E) where family processes moderate genetic effects via diathesis-stress or differential-susceptibility frameworks (29, 30), and gene-environment correlations (rGE) where family processes serve as mediating mechanisms linking genetic factors to adolescent externalizing behaviors (7). There are evidence supporting both G×E and rGE involving family processes. For example, parental monitoring buffered the association between externalizing disorders PRS and adolescent externalizing behaviors among White adolescents from a high-risk sample enriched for alcohol use disorders (31). Adolescents’ PRS for externalizing problems predicted greater family dysfunction among White youth after controlling for maternal and paternal PRS, suggesting evocative rGE, although the effect was very small (32). Moreover, White, Black, and Hispanic youth with genetic predispositions toward externalizing behaviors may be more likely to experience decreased parental acceptance, as adolescents’ behavioral problems may negatively influence the parent-child relationship (16, 33–35). Understanding multiple forms of gene-environment interplay is important to better understand how genetic and family processes function together to influence adolescent externalizing behaviors over time.

### 1.4 Studying gene-environment interplay in diverse populations

The vast majority of genetically informed research has focused on White populations of European ancestry, limiting our understanding of gene-environment interplay among racially and ethnically diverse groups. The accuracy and clinical utility of PRS can vary significantly across racial and ethnic groups (36). When PRS are primarily developed from findings derived from participants of European-ancestry, their predictability may decrease in diverse populations due to variations in both allele frequencies and genetic variant effect sizes, and differences in relevant sociocultural contexts (e.g., discrimination experiences) that can also influence genetic effect sizes. The historical focus on non-Hispanic White individuals can obscure how genetic and environmental pathways of risk and resilience differ across diverse populations. This lack of accuracy can result in disparities in identifying at-risk individuals and tailoring interventions, ultimately exacerbating existing racial and ethnic health disparities (18). As such, lack of understanding of genetic influences among minority populations may result in insufficient resources and support for those who need it the most.

Moreover, environmental factors, such as family processes, may interact with genetic factors in complex ways that differ across racial-ethnic and cultural groups. The effect of parenting behaviors and family environment on adolescent externalizing outcomes may vary across racial-ethnic groups. Certain aspects of parenting, such as parental warmth, have been shown to have positive behavioral effects across Asian, African, European, North American, and South American cultural groups (27). However, cultural differences can underlie significant variations in parenting values, parenting norms, and parental effects on child development (37, 38). To develop effective prevention and intervention strategies, it is essential to study gene-environment interplay among diverse populations as a broader range of genetic and environmental data will enhance our understanding of the unique pathways influencing externalizing behaviors and inform family-based interventions to be culturally sensitive and effective across racial-ethnic groups.

### 1.5 The current study

Guided by theories of developmental psychopathology and building on prior theoretical frameworks that emphasize taking a developmental approach to study gene-environment interplay (7), the overarching aims of the current study were to 1) identify distinct trajectories of externalizing behaviors from late childhood to early adolescence, 2) examine how genetic risk predicts developmental trajectories of externalizing behaviors, and 3) investigate the role of family processes in moderating and mediating genetic effects on trajectories of externalizing behaviors (G×E and rGE). To address these aims, we used a PRS approach to examine the effects of both adult-based and child-based PRS related to externalizing behaviors. We examined multiple family processes that have been shown to have a robust effect on adolescent externalizing behaviors, namely parental monitoring, parental acceptance, and family conflict, in order to examine their unique effects. We examined these gene-environment interplay processes among Hispanic, Black, and White youth.

We hypothesized that distinct trajectories of externalizing behaviors would be identified: the majority of youth would follow a low-risk trajectory characterized by zero or low externalizing behaviors, a proportion of youth would follow trajectories characterized by higher risk of externalizing behaviors, such as an increasing trajectory or a persistent high externalizing trajectory (Hypothesis 1). We further hypothesized that both adult-based and child-based PRS would predict trajectories of externalizing behaviors, and higher PRS would predict higher likelihood of membership in trajectories that represent higher risk (Hypothesis 2). Higher family conflict would be associated with higher likelihood of following high risk externalizing trajectories, whereas higher parental acceptance and parental monitoring would be associated with lower likelihoods of following high risk externalizing trajectories (Hypothesis 3). In terms of gene-environment interplay, we hypothesized that family conflict would exacerbate the effects of PRS, whereas parental acceptance and monitoring would attenuate or buffer the effects of PRS (Hypothesis 4). We further hypothesized that effects of PRS may be partially mediated by family processes, such that higher PRS would be associated with higher family conflict and lower parental acceptance and monitoring, which in turn would be associated with higher likelihood of following high risk externalizing trajectories (Hypothesis 5).

Because PRS were derived from GWAS conducted with primarily White individuals of European descent, which may be biased and have limited predictive power in non-European samples, we hypothesized that effects of PRS would be smaller or even non-significant in the Black and Hispanic subsamples. Hypotheses and analyses were preregistered in OSF (https://osf.io/9p5vk).

## 2 Materials and methods

### 2.1 Sample and procedures

Data were drawn from the Adolescent Brain Cognitive Development (ABCD) Study (39). The ABCD study is an ongoing study that enrolled youth ages 9-10 years old and follows them for approximately 10 years across adolescence. A total of 11,875 adolescents were assessed at baseline, with follow up assessments conducted annually. Participants were primarily recruited from schools across 21 sites in the U.S., with some participants recruited through community events and referral systems (40). The ABCD study collects rich data on biological, neurocognitive, behavioral, and environmental measures from the participants. These data were made available to qualified researchers through annual data releases via the National Institute of Mental Health Data Archive. Data from the baseline (T1, 9/10 years old), 2-year follow up (T3, 11/12 years old), and 3-year follow up (T4, 12/13 years old) assessments were used. We included youth who had genetic data available for the calculating of polygenic scores and data on externalizing behaviors. The analytic sample focused on youth who were identified as non-Hispanic White (n = 5,907, 53.0% male), non-Hispanic Black (n = 1,694, 49.6% male), or Hispanic (n = 2,117, 53.4% male) by parent report at baseline, the three largest racial-ethnic groups in the ABCD sample for which there are sufficient sample sizes for within-group analyses.

### 2.2 Measures

#### 2.2.1 Externalizing behaviors

Parents completed the reliable and well-validated Child Behavior Checklist (CBCL) (41) at baseline, 1-year follow up, 2-year follow up, and 3-year follow up assessments. The CBCL asks parents to report on their child’s psychopathology and includes 8 subscales: anxious/depressed, withdrawn/depressed, somatic complaints, social problems, thought problems, attention problems, rule-breaking behavior, and aggressive behavior. Parents were asked to report on 112 items (e.g., “my child gets in many fights” and “my child is impulsive or acts without thinking”) and assess the degree to which they believed the item applied to their child from *not true* (scored as 0) to *very true/often true* (scored as 2). The parents were asked to consider their child’s behavior during the past 6 months when assessing the relevance of the items. The CBCL provides Externalizing Composite t-scores that combine the rule-breaking and aggressive behavior scales. Higher composite scores indicate higher levels of externalizing behaviors and problems. Cronbach’s alpha for the externalizing behaviors measure ranged from.89 to.92 across waves and racial-ethnic groups.

#### 2.2.2 Family conflict

Adolescents completed nine items from the Family Conflict subscale of the Moos Family Environment Scale (FES) at baseline, which assessed the amount of openly expressed conflict among family members (42). Participants indicated whether statements about conflict in the family were true or false in their home environment (e.g., “we fight a lot in our family”). Items were scored either 1 or 0 (i.e., true or false) with appropriate reverse coding for certain items (e.g., “family members hardly ever lose their temper”). Scores were summed across items. Prorated scores were calculated by multiplying the raw scores by the total number of items and dividing by the number of items completed by the participant. If a participant answered less than 5 items, their scores were not counted and coded as missing. Higher scores indicated more conflict within the family environment. This scale has acceptable internal consistency: Cronbach’s alpha was.69,.65, and.65 for White, Black, and Hispanic youth, respectively.

#### 2.2.3 Parental acceptance

At the baseline assessment, adolescents were asked to complete a subscale of the Child Report of Behavior Inventory (CRPBI) (43, 44) that measured their perceptions of their caregiver’s warmth, acceptance, and responsiveness (e.g., “my caregiver makes me feel better after talking over my worries with him/her”). The ABCD study utilized five items with the highest factor loadings from the original 10-item scale (45). Participants were responded to items related to their perceived levels of acceptance from their two primary caregivers. They reported the extent to which they agreed with each item based on a scale ranging from 1 (*not at all*) to 3 (*very much*). A total parental acceptance score was calculated by averaging the scores on the five items across the two caregivers. If scores on only one primary caregiver was provided, those scores were used to indicate parental acceptance. Cronbach’s alpha for the measure was.71,.72, and.67 for White, Black, and Hispanic youth, respectively.

#### 2.2.4 Parental monitoring

At the baseline assessment, adolescents completed the Parental Monitoring Scale which assessed parental monitoring and knowledge of their children’s whereabouts and who their children were spending time with (45). The scale consists of five items (e.g., “how often do your parents/guardians know where you are?” and “how often do your parents know who you are with when you are not at school and away from home?”). Participants were asked to indicate the extent to which they agreed with each item based on a scale ranging from 1 (*not at all*) to 5 (*very often*). Total scores were calculated by averaging the individual’s responses across all five items.

#### 2.2.5 Genotyping and genome-wide polygenic scores

Saliva samples from youth were collected at the baseline visit and shipped from the collection site to Rutgers University Cell and DNA Repository (RUCDR) for genotyping. The Smokescreen Genotyping Array (46) was used for genotyping. RUCDR performed DNA quality controls based on calling signals and variant call rates, and the quality-controlled genotyping data contains 11,099 unique individuals with 516,598 genetic variants in the ABCD study. Imputation was performed via the TOPMed imputation server using mixed ancestry and Eagle v2.4 phasing. Single nucleotide polymorphisms (SNPs) with a genotyping rate < 0.95 or that violated Hardy–Weinberg equilibrium (p < 10^-6^) or with minor allele frequency < 0.01 were excluded from analysis.

We calculated two genome-wide polygenic scores based on estimates from two GWAS studies. First, polygenic scores for adult externalizing (AdultExt-PRS) were calculated using estimates from a GWAS of adult externalizing behaviors (47). This GWAS was conducted with a sample of about 1.5 million people of European ancestry and used multivariate genomic structural equation modeling to capture genetic influences on a number of externalizing-related traits (e.g., substance use disorders, antisocial behaviors). Second, polygenic scores for childhood aggression (ChildAgg-PRS) were calculated using estimates from a GWAS of childhood aggression based on a total of 328,935 observations from 87,485 children of European ancestry (48). Past research has shown utility of polygenic risk scores derived from these GWAS in predicting behavioral traits in Hispanic and Black participants (16, 17, 21, 49). Polygenic scores were calculated using the PRS-CS method (50). To account for population stratification, residualized polygenic scores that account for the first 10 genetic ancestry principal components were calculated. These residualized polygenic scores were standardized for subsequent data analyses.

#### 2.2.6 Covariates

Adolescents’ age in years, sex assigned at birth, parental education, and parental report of family income were considered as control variables.

### 2.3 Analytic strategy

Preliminary analyses were conducted to examine descriptive statistics and bivariate correlations between study variables. Attrition and missing data patterns were also examined. All analyses were conducted separately by racial/ethnic group due to potential racial/ethnic variations in externalizing problem developmental trajectories, parenting/family processes, and polygenic utility (9, 36–38). To identify distinct trajectories of externalizing behaviors, we conducted growth mixture modeling (GMM) using Mplus. Both linear and quadratic growth were examined. A series of models ranging from 2 to 6 latent classes were examined, and the optimal model was determined based on model fit indices (i.e., the Lo-Mendell-Rubin Likelihood Ratio Test (LMR-LRT), Akaike Information Criterion (AIC), sample-size-adjusted BIC (aBIC), entropy). Lower AIC and aBIC indicate better model fit. Higher entropy indicates better classification utility. A significant LMR-LRT indicates the model fit is significantly better than the model with one fewer class. Model interpretability (e.g., class size and meaningfulness of each class) were considered, along with model fit indices, to determine the optimal model.

After identifying trajectories of externalizing behaviors using GMM, a series of multinomial logistic regression models were conducted to examine how polygenic scores, family processes, and their interactions (G×E) predict the likelihood of following different trajectories, using the R3STEP command in Mplus, an automatic approach linking covariates to class memberships (51). We started with a model examining main effects of AdultExt-PRS and ChildAgg-PRS, controlling for age, sex, parental education, and family income (Step 1). Next, we added family conflict, parental acceptance, and parental monitoring to the model in order to examine their main effects (Step 2). Finally, we evaluated the interaction effects between AdultExt-PRS and ChildAgg-PRS and family processes by adding product terms between polygenic scores and mean-centered family variables to the model (Step 3). Interaction effects were tested separately for each family variable to avoid multicollinearity. Statistically significant interaction effects were followed up by robustness analysis where interaction terms between covariates and the polygenic score and family variable are added to the model in order to account for potential confounding effects (52).

In order to examine rGE, we extracted latent class membership from the GMM models. Because indirect effects with nominal variables cannot be estimated in Mplus, we created dummy-coded variables to reflect membership in each trajectory of externalizing behaviors and included class membership for high and moderate externalizing trajectories as binary outcome variables with the low decreasing trajectory as the reference group (53). Path models were conducted where AdultExt-PRS and ChildAgg-PRS were specified as predictors of family conflict, parental acceptance, and parental monitoring, which in turn were specified to predict class membership of externalizing trajectories. Direct associations between AdultExt-PRS and ChildAgg-PRS and class membership were also specified. AdultExt-PRS and ChildAgg-PRS were specified to be correlated, as well as the different family process variables. Age, sex, parental education, and family income were specified as covariates for latent class membership. Significant indirect effects from AdultExt-PRS and ChildAgg-PRS to class membership for externalizing trajectories via family processes were evaluated using bias-corrected bootstrapping with a 95% confidence interval (CI) (54). CIs not including zero would indicate statistically significant indirect effects.

Analyses accounted for clustering within study sites and family using the stratification and cluster commands in Mplus. Missing data were accounted for by multiple imputation (55). Because we tested six G×E effects between two PRS and three family processes, we used a conservative Bonferroni corrected *p* value (*p* <.008) to correct for multiple testing when evaluating statistical significance of the G×E effects, in order to reduce the possibility of false positive findings. Other coefficients were evaluated using *p* <.05.

## 3 Results

### 3.1 Preliminary analysis

Among the whole sample, 0.2% of participants had missing data at T1 across externalizing problems, family conflict, parental monitoring, or parental acceptance. Proportions of the sample with missing data increased at each follow-up assessments (T2, 5.4%; T3, 8.0%; T4, 14.6%). Participants with missing data at T1 had significantly higher externalizing problem at T2, T3, and T4 and lower parental education. Participants with missing data at T2 had significantly lower household income, parental education, higher family conflict, and greater externalizing problems at T1 and T3. Participants with missing data at T3 were more likely to be female, had significantly lower household income, parental education, and significantly higher externalizing problems at T1 and T4. Participants with missing data at T4 were more likely to be female, had higher family conflict, lower parental education and family income, and greater externalizing problems at T1, T2, and T3. Participants with missing data at T2, T3, or T4 were more likely to be Black or Hispanic than White.

Descriptive statistics and bivariate correlations between study variables for each racial-ethnic subgroup are presented in **Table 1**. AdultExt-PRS and ChildAgg-PRS were modestly positively correlated with small correlations across racial/ethnic groups. Both AdultExt-PRS and ChildAgg-PRS were positively correlated with adolescent externalizing behaviors from T1 to T4 across racial-ethnic groups. Family conflict was positively correlated with externalizing behaviors across assessments and racial/ethnic groups. Parental acceptance and parental monitoring were generally negatively correlated with externalizing behaviors, with some correlations being very small and non-significant.

**Table 1:** Descriptive statistics and bivariate correlations between study variables across racial/ethnic groups.

White Youth	1	2	3	4	5	6	7	8	9	10	11	12	13
1. Age	–												
2. Sex	**.03**	–											
3. Family Income	**.03**	-.02	–										
4. Parent Education	-.01	-.02	**.51**	–									
5. AdultExt-PRS	.00	-.01	**-.16**	**-.19**	–								
6. ChildAgg-PRS	-.03	-.01	**-.09**	**-.11**	**.22**	–							
7. Family Conflict	**-.05**	**.06**	**-.13**	**-.11**	**.06**	**.05**	–						
8. Parental Acceptance	**.03**	**-.06**	**.09**	**.08**	**-.06**	**-.04**	**-.34**	–					
9. Parental Monitoring	**.11**	**-.18**	**.14**	**.10**	**-.04**	-.02	**-.26**	**.38**	–				
10. Externalizing T1	-.02	**.11**	**-.22**	**-.15**	**.14**	**.12**	**.20**	**-.13**	**-.14**	–			
11. Externalizing T2	-.02	**.11**	**-.21**	**-.15**	**.16**	**.10**	**.17**	**-.14**	**-.12**	**.76**	–		
12. Externalizing T3	.01	**.11**	**-.18**	**-.12**	**.15**	**.10**	**.15**	**-.13**	**-.10**	**.70**	**.74**	–	
13. Externalizing T4	-.01	**.10**	**-.17**	**-.11**	**.16**	**.10**	**.14**	**-.13**	**-.11**	**.65**	**.70**	**.76**	–
Mean	119	.53	8.2	17.4	-.03	-.02	2.0	4.4	2.7	4.2	4.0	3.8	3.9
SD	7.5	.50	1.7	1.9	1.1	1.1	2.0	.47	.29	5.5	5.4	5.3	5.4
Black Youth	1	2	3	4	5	6	7	8	9	10	11	12	13
1. Age	–												
2. Sex	.02	–											
3. Family Income	.04	-.01	–										
4. Parent Education	**.05**	.03	**.58**	–									
5. AdultExt-PRS	.01	**-.06**	**-.09**	-.02	–								
6. ChildAgg-PRS	-.04	-.02	-.02	-.03	**.15**	–							
7. Family Conflict	**-.07**	**.08**	**-.09**	**-.09**	.00	.01	–						
8. Parental Acceptance	-.04	**-.07**	-.03	-.04	.00	.00	**-.23**	–					
9. Parental Monitoring	**.06**	**-.16**	-.01	-.01	.01	-.01	**-.20**	**.37**	–				
10. Externalizing T1	.01	**.14**	**-.14**	**-.07**	**.07**	**.05**	**.12**	-.04	**-.06**	–			
11. Externalizing T2	.02	**.11**	**-.13**	**-.06**	**.08**	**.05**	**.12**	-.02	**-.06**	**.73**	–		
12. Externalizing T3	.01	.04	**-.10**	.00	**.12**	**.08**	**.08**	-.03	**-.05**	**.65**	**.71**	–	
13. Externalizing T4	.01	.02	**-.07**	.00	**.15**	**.06**	**.09**	**-.09**	**-.07**	**.64**	**.65**	**.70**	–
Mean	119	.50	5.0	15.0	-.03	.01	2.5	4.3	2.7	5.3	4.9	4.1	4.0
SD	7.28	.50	2.64	2.37	.76	.86	2.03	.59	.30	7.0	6.7	6.1	6.0
Hispanic Youth	1	2	3	4	5	6	7	8	9	10	11	12	13
1. Age	–												
2. Sex	.01	–											
3. Family Income	.04	-.01	–										
4. Parent Education	-.02	.01	**.55**	–									
5. AdultExt-PRS	.00	.02	-.03	-.02	–								
6. ChildAgg-PRS	-.00	-.01	**-.05**	-.02	**.18**	–							
7. Family Conflict	**-.06**	**.05**	**-.09**	**-.08**	.02	.01	–						
8. Parental Acceptance	**.04**	**-.06**	.03	**.05**	.02	-.00	**-.29**	–					
9. Parental Monitoring	**.09**	**-.20**	**.10**	**.10**	.02	.02	**-.22**	**.38**	–				
10. Externalizing T1	**-.05**	**.11**	**-.11**	-.04	**.09**	**.07**	**.13**	**-.11**	**-.08**	–			
11. Externalizing T2	**-.06**	**.10**	**-.11**	-.01	**.07**	**.09**	**.14**	**-.09**	-.04	**.72**	–		
12. Externalizing T3	-.04	**.09**	**-.09**	.00	**.09**	**.07**	**.07**	**-.09**	-.04	**.65**	**.68**	–	
13. Externalizing T4	-.04	**.12**	-.04	.04	**.06**	**.07**	**.07**	**-.06**	-.05	**.57**	**.62**	**.68**	–
Mean	119	.53	6.3	14.8	.05	.04	2.0	4.4	2.7	4.5	4.2	4.1	3.8
SD	7.57	.50	2.4	3.21	.965	.984	1.9	.54	.29	5.6	5.3	5.5	5.2
**Mean differences**	W > B H > B	W > B H > B	W >H > B	W > H W >B	–	–	B > W B > H	ns	W > H > H	B > W B > H	B > W B > H	ns	ns

W, White; B, Black; H, Hispanic; ns, nonsignificant. Coefficients with *p* <.05 are bolded.

### 3.2 Identifying trajectories of externalizing behaviors

**Figure 1:**
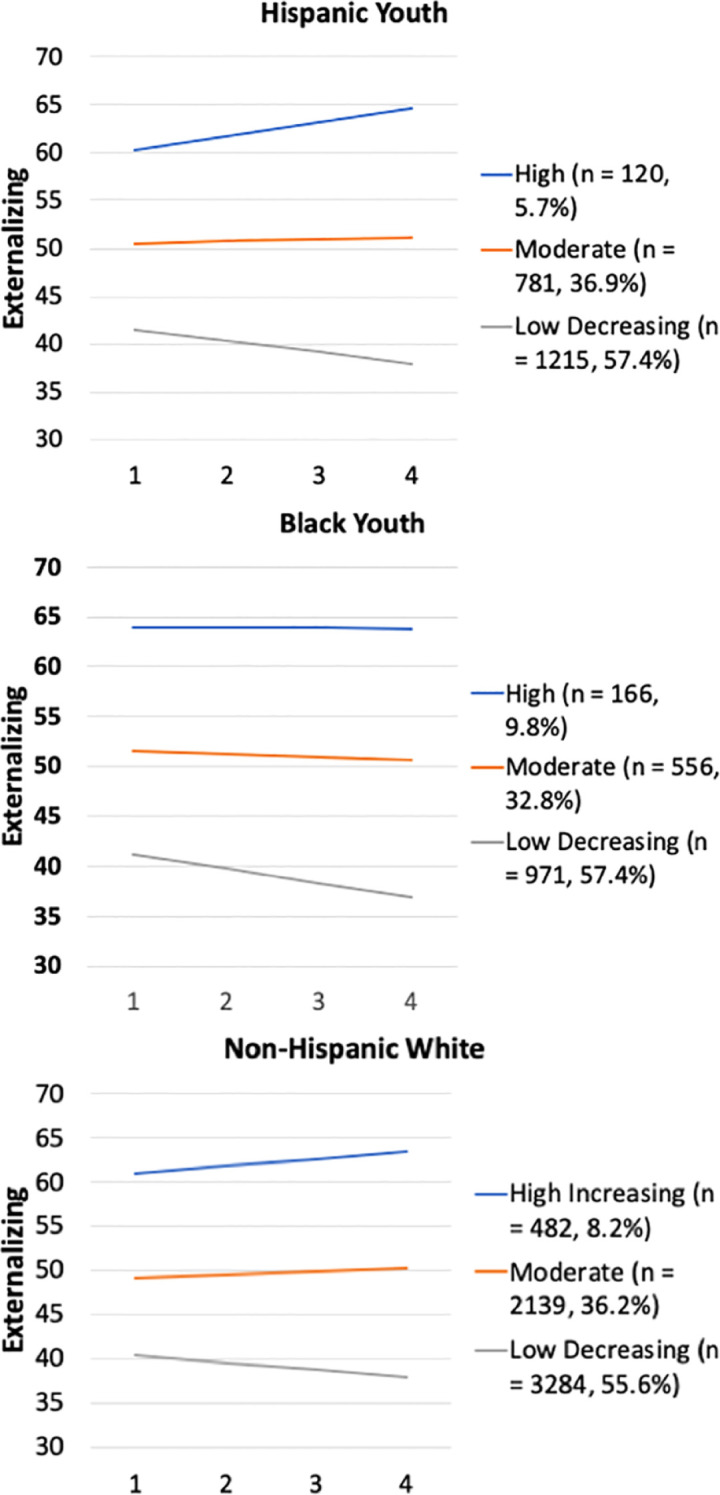
Trajectories of externalizing behaviors among non-Hispanic white, non-Hispanic black, and Hispanic youth. X axis represents the assessment wave.

Unconditional latent growth curve models examining linear and quadratic slopes indicated that the quadratic slope was either not significant (for Black and Hispanic youth) or lacked significant variance (for White youth) across groups. Thus, we proceeded with only examining linear slope in GMM models. A series of GMM models specifying two to six latent classes of externalizing behavior trajectories were evaluated. Fit indices for the models were presented in **Table 2**. The 3-class model yielded the best fit across the three racial-ethnic groups. The identified trajectories of externalizing behaviors were also largely similar across groups, with slight differences in the proportion of youth following each trajectory (see **Figure 1**). For White youth, 55.6% followed a *Low Decreasing* trajectory characterized by low levels of externalizing behaviors that decreased over time; 36.2% of the sample followed a *Moderate* trajectory of externalizing behaviors, characterized by moderate levels of externalizing behaviors that were stable over time; and 8.2% of the sample followed a *High Increasing* trajectory, characterized by highest levels of externalizing behaviors that increased over time. For Black youth, 57.4% followed a *Low Decreasing* trajectory, 32.8% followed a *Moderate* trajectory, and 9.8% followed a *High* trajectory of externalizing problems. For Hispanic youth, 57.4% followed a *Low Decreasing* trajectory, 36.9% followed a *Moderate* trajectory, and 5.7% followed a *High* trajectory of externalizing problems.

**Table 2:** Fit indices for growth mixture models of externalizing behaviors across racial/ethnic groups.

White Youth				
Number of Classes	AIC	aBIC	LMR LRT p value	Entropy
1	151937.39	151968.95	–	–
2	151366.74	151408.81	.0038	.703
**3**	**150975.56**	**151028.16**	**.0070**	**.756**
4	150702.93	150766.04	.2311	.768
5	150708.93	150782.56	.5000	.678
6	150552.87	150637.02	.5413	.761
Black Youth				
Number of Classes	AIC	aBIC	LMR LRT p value	Entropy
1	41854.84	41875.17	–	–
2	41625.41	41652.51	.0722	.703
**3**	**41447.59**	**41481.46**	**.0488**	**.789**
4	41391.18	41431.82	.3199	.752
5	41365.90	41413.31	.5635	.762
6	41322.68	41376.88	.6016	.724
Hispanic Youth				
Number of Classes	AIC	aBIC	LMR LRT p value	Entropy
1	53007.57	53029.89	–	–
2	52824.71	52854.47	.1143	.670
**3**	**52708.54**	**52745.75**	**.0207**	**.759**
4	52595.74	52640.39	.0785	.764
5	52547.58	52599.68	.7076	.751
6	52559.69	52619.23	.3377	.802

The selected best-fitting models are bolded.

### 3.3 Main effects of polygenic scores and family processes

Associations between PRS, family processes, and externalizing trajectories are presented in **Tables 3**–**5**. Among White youth (see **Table 3**), after accounting for the effects of age, sex, family income, and parent education, both AdultExt-PRS and ChildAgg-PRS were associated with higher risk of following the High Increasing and Moderate trajectories compared to the Low Decreasing trajectory of externalizing problems. AdultExt-PRS was also significantly associated with higher likelihood of following the High Increasing trajectory compared to the Moderate trajectory. Higher levels of family conflict were associated with higher likelihood of following the High Increasing and Moderate trajectories compared to the Low Decreasing trajectory of externalizing problems, whereas higher levels of parental acceptance were associated with lower likelihood of following the High Increasing and Moderate trajectories compared to the Low Decreasing trajectory of externalizing problems. High family conflict was also associated with greater likelihood of following the High Increasing trajectory compared to the Moderate trajectory. Parental monitoring was not significantly associated with trajectories of externalizing problems in our models where family conflict and parental acceptance were considered.

**Table 3:** Coefficients from multinomial logistic regression models predicting trajectories of externalizing behaviors among non-Hispanic white youth.

	High Increasing vs Low Decreasing		Moderate vs Low Decreasing		High Increasing vs Moderate	
**Step 1**	OR	95% CI	OR	95% CI	OR	95% CI
Age	.99	.98, 1.01	.99*	.98,.996	1.01	.99, 1.03
Sex	1.59**	1.25, 2.02	1.32**	1.14, 1.53	1.20	.92, 1.57
Parent Education	.94*	.89, 1.00	.97	.93, 1.01	.97	.90, 1.04
Family Income	.77***	.71,.82	.92**	.88,.98	.83***	.77,.89
AdultExt-PRS	1.49***	**1.33, 1.68**	1.23***	**1.15, 1.32**	1.22**	**1.07, 1.38**
ChildAgg-PRS	1.26***	**1.13, 1.42**	1.16***	**1.08, 1.25**	1.09	.96, 1.23
**Step 2**						
Family Conflict	1.24***	**1.17, 1.32**	1.09***	**1.05, 1.14**	1.13***	**1.06, 1.21**
Parental Acceptance	.56***	**.36,.87**	.67**	**.51,.89**	.83	.52, 1.33
Parental Monitoring	.81	.62, 1.05	.97	.82, 1.15	.83	.62, 1.11
**Step 3**						
AdultExt-PRS x Family Conflict	1.00	.95, 1.06	1.00	.96, 1.04	1.00	.94, 1.06
AdultExt-PRS x Parental Acceptance	1.49	.98, 2.26	1.04	.80, 1.37	1.43	.93, 2.20
AdultExt-PRS x Parental Monitoring	.87	.67, 1.13	1.00	.86, 1.17	.87	.66, 1.15
ChildAgg-PRS x Family Conflict	.99	.93, 1.04	1.04	1.00, 1.08	.95	.89, 1.01
ChildAgg-PRS x Parental Acceptance	.95	.61, 1.49	1.17	.89, 1.55	.81	.50, 1.31
ChildAgg-PRS x Parental Monitoring	1.21	.93, 1.57	.92	.78, 1.09	1.31	.99, 1.74

AdultExt-PRS, polygenic risk scores for adult externalizing; ChilAgg-PRS, polygenic risk scores for childhood aggression. Significant main effects of PRS and family processes are bolded. OR, odds ratio. CI, confidence intervals. *p <.05, **p <.01, ***p <.001.

**Table 4:** Coefficients from multinomial logistic regression models predicting trajectories of externalizing behaviors among non-Hispanic black youth.

	High vs Low Decreasing		Moderate vs Low Decreasing		High vs Moderate	
**Step 1**	OR	95% CI	OR	95% CI	OR	95% CI
Age	1.01	.98, 1.03	1.01	.99, 1.03	1.00	.97, 1.03
Sex	1.45	.97, 2.17	1.11	.85, 1.44	1.31	.82, 2.08
Parent Education	1.03	.92, 1.16	1.01	.94, 1.08	1.02	.89, 1.18
Family Income	.89*	.80,.98	1.00	.97, 1.07	.89	.79,.99
AdultExt-PRS	1.76**	**1.32, 2.35**	1.16	.97, 1.39	1.52*	**1.09, 2.12**
ChildAgg-PRS	1.23	.98, 1.55	1.04	.89, 1.22	1.19	.91, 1.55
**Step 2**						
Family Conflict	1.14**	**1.04, 1.26**	1.02	.96, 1.10	1.12	1.00, 1.25
Parental Acceptance	.81	.37, 1.80	.56**	**.35,.91**	1.45	.58, 3.64
Parental Monitoring	.80	.57, 1.14	1.17	.90, 1.52	.69*	**.45, 1.05**
**Step 3**						
AdultExt-PRS x Family Conflict	1.01	.88, 1.15	.95	.87, 1.05	1.06	.90, 1.24
AdultExt-PRS x Parental Acceptance	1.12	.43, 2.89	1.06	.55, 2.06	1.05	.35, 3.15
AdultExt-PRS x Parental Monitoring	1.04	.71, 1.53	1.05	.76, 1.44	.99	.63, 1.58
ChildAgg-PRS x Family Conflict	.94	.84, 1.05	1.03	.94, 1.12	.92	.80, 1.05
ChildAgg-PRS x Parental Acceptance	.79	.35, 1.79	.86	.47, 1.59	.92	.34, 2.47
ChildAgg-PRS x Parental Monitoring	1.29	.92, 1.82	1.26	.92, 1.74	1.02	.66, 1.59

AdultExt-PRS, polygenic risk scores for adult externalizing; ChilAgg-PRS, polygenic risk scores for childhood aggression. Significant main effects of PRS and family processes are bolded. OR, odds ratio. CI, confidence intervals. *p <.05, **p <.01, ***p <.001,

**Table 5:** Coefficients from multinomial logistic regression models predicting trajectories of externalizing behaviors among Hispanic youth.

	High vs Low Decreasing		Moderate vs Low Decreasing		High vs Moderate	
**Step 1**	OR	95% CI	OR	95% CI	OR	95% CI
Age	.98	.95, 1.01	.99	.97, 1.01	.99	.95, 1.02
Sex	1.32	.81, 2.15	1.46**	1.15, 1.86	.90	.53, 1.54
Parent Education	1.15*	1.02, 1.28	1.05	1.00, 1.10	1.10	.97, 1.24
Family Income	.83**	.74,.93	.93*	.87,.99	.89	.78, 1.02
AdultExt-PRS	1.24	0.98, 1.57	1.06	.93, 1.19	1.18	.91, 1.52
ChildAgg-PRS	1.33*	**1.04, 1.72**	1.18*	**1.04, 1.33**	1.13	.86, 1.49
**Step 2**						
Family Conflict	.99	.86, 1.14	1.09*	**1.02, 1.17**	.90	.77, 1.06
Parental Acceptance	.50	.23, 1.09	.69	.43, 1.11	.72	.31, 1.70
Parental Monitoring	.91	.52, 1.59	1.08	.84, 1.39	.84	.45, 1.55
**Step 3**						
AdultExt-PRS x Family Conflict	1.04	.90, 1.21	1.07	.99, 1.15	.98	.83, 1.15
AdultExt-PRS x Parental Acceptance	1.50	.59, 3.86	.96	.55, 1.66	1.57	.57, 4.36
AdultExt-PRS x Parental Monitoring	.88	.49, 1.57	1.05	.79, 1.38	.84	.44, 1.60
ChildAgg-PRS x Family Conflict	.98	.84, 1.13	1.03	.97, 1.11	.95	.80, 1.12
ChildAgg-PRS x Parental Acceptance	.86	.31, 2.34	1.08	.68, 1.71	.79	.26, 2.41
ChildAgg-PRS x Parental Monitoring	1.02	.50, 2.05	1.10	.87, 1.40	.92	.42, 2.00

AdultExt-PRS, polygenic risk scores for adult externalizing; ChilAgg-PRS, polygenic risk scores for childhood aggression. Significant main effects of PRS and family processes are bolded. OR, odds ratio. CI, confidence intervals. *p <.05, **p <.01, ***p <.001, ^+^ *p* = .106

Among Black youth (see **Table 4**), similar to the findings for White youth, AdultExt-PRS was associated with higher risk of following the High trajectory compared to the Low Decreasing or Moderate trajectory. Higher levels of family conflict were associated with higher likelihood of following the High trajectory compared to the Low Decreasing trajectory of externalizing problems, whereas higher levels of parental acceptance were associated with lower likelihood of following the Moderate trajectory compared to the Low Decreasing trajectory of externalizing problems. Higher parental monitoring was associated with lower likelihood of following the high externalizing trajectory relative to the moderate externalizing trajectory. However, contrary to the findings for White youth, ChildAgg-PRS was not significantly associated with trajectories of externalizing problems.

Among Hispanic youth (see **Table 5**), ChildAgg-PRS was associated with higher likelihood of following the High and Moderate trajectories compared to the Low Decreasing trajectory of externalizing problems. However, contrary to the findings for White and Black youth, AdultExt-PRS was not significantly associated with trajectories of externalizing problems. Higher levels of family conflict were associated with greater likelihood of following the Moderate trajectory compared to the Low Decreasing trajectory. However, parental acceptance and parental monitoring were not significantly associated with trajectories of externalizing problems.

### 3.4 Interactions between polygenic scores and family processes

Across White, Black, and Hispanic subgroups, no significant G×E effects (based on the *a priori* Bonferroni corrected *p* value of.008) were detected when interaction effects were examined simultaneously for family conflict, parental acceptance, and parental monitoring (see **Tables 3**-**5**, Step 3). Sensitivity analyses were conducted to examine interaction effects between polygenic scores and each family variable in separate models; no significant G×E effects were found in these sensitivity analyses.

### 3.5 Examining rGE as mechanisms of polygenic influences

**Figure 2:**
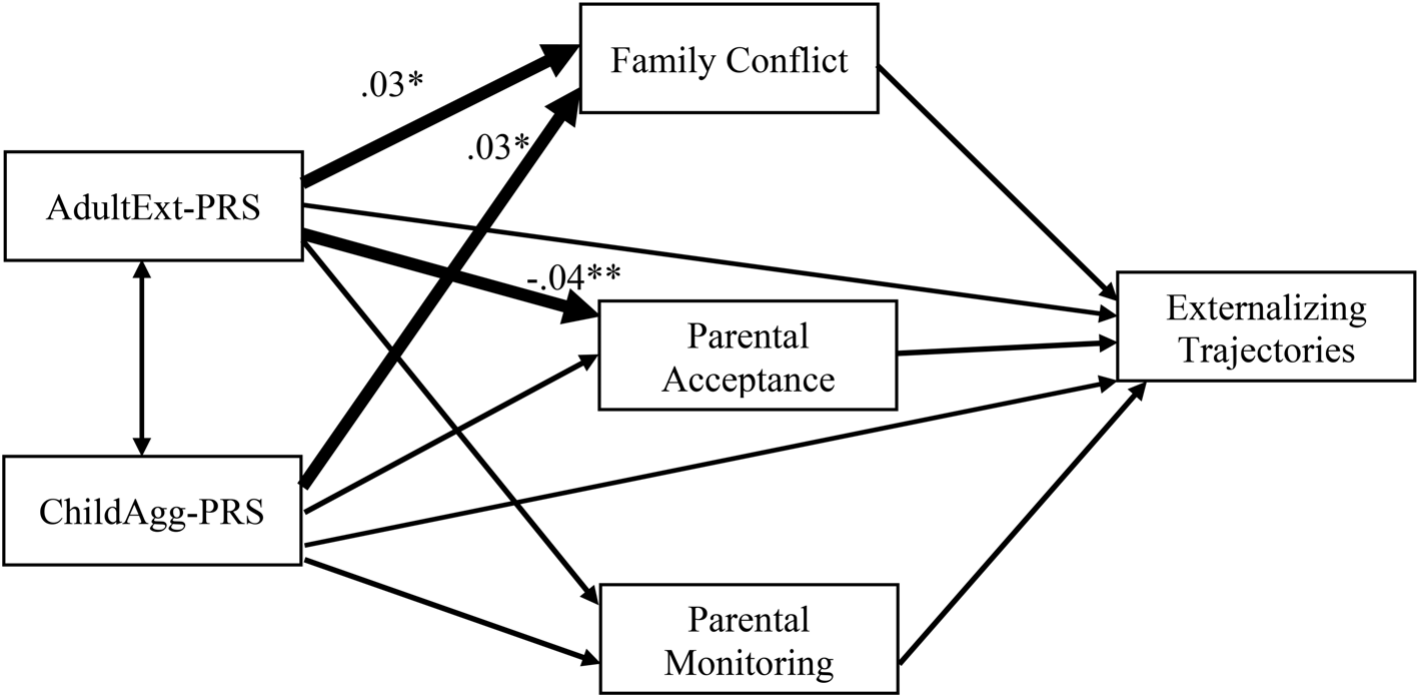
Path Model Examining rGE linking polygenic risk scores and family processes to trajectories of externalizing behaviors. AdultExt-PRS = polygenic risk scores for adult externalizing; ChilAgg-PRS = polygenic risk scores for childhood aggression. Statistically significant standardized coefficients linking polygenic scores to family processes (representing rGE) are presented for White youth. No significant rGE was found for Black and Hispanic youth. Coefficients for other paths are not presented in the figure for ease of presentation but are available in **Supplementary Tables 2**-**4**.

Among White youth (see **Figure 2**; **Supplementary Table 2**), both AdultExt-PRS and ChildAgg-PRS were associated with higher family conflict, which in turn were associated with greater likelihood of following trajectories of higher externalizing problems. In addition, AdultExt-PRS was associated with lower parental acceptance, which in turn was associated with greater risk of following the High Increasing and Moderate trajectories compared to the Low Decreasing trajectory of externalizing problems. 95% CIs indicated a significant indirect effect linking ChildAgg-PRS to the High Increasing trajectory compared to the Low Decreasing Trajectory via family conflict. Contrary to findings for White youth, no significant rGE effects were detected among Black (**Supplementary Table 3**) and Hispanic youth (**Supplementary Table 4**).

## 4 Discussion

The current study took a developmental approach to examine the interplay between polygenic risk and family processes in predicting trajectories of externalizing behaviors from late childhood to early adolescence among racially-ethnically diverse youth. Findings revealed heterogeneity in developmental trajectories of externalizing behaviors among racially-ethnically diverse adolescents. The findings contribute to the literature by demonstrating that both polygenic risk for adult externalizing behaviors and polygenic risk for childhood aggression uniquely predict trajectories of externalizing behaviors. Findings also highlight the important role of family processes, especially family conflict, in influencing adolescent externalizing behaviors and mediating polygenic influences on trajectories of externalizing behaviors.

Consistent with our Hypothesis 1 and prior research, results indicated substantial heterogeneity in developmental trajectories of externalizing behaviors over time. Across all racial-ethnic subgroups examined in our study, the majority of youth followed a trajectory characterized by low levels of externalizing behaviors that decreased over time. It is notable that youth who followed trajectories with higher levels of externalizing behaviors did not show significant decline in externalizing behaviors during this developmental period (age 9/10 – age 12/13). Prior research showed that externalizing behaviors generally decline from early childhood through adolescence, with heterogeneity in trajectories of externalizing behaviors over time (56). We extend prior findings to show that decline in externalizing behaviors from late childhood to early adolescence is apparent only for adolescents who exhibit relatively lower levels of externalizing behaviors. Levels of externalizing behaviors tend to be stable or even increase over time among adolescents who exhibited higher levels of externalizing behaviors. This highlights the critical need for intervention efforts that target individuals with high externalizing problems in late childhood as these externalizing problems may persist over time.

Consistent with our Hypothesis 2, both AdultExt-PRS and ChildAgg-PRS predicted higher likelihood of following trajectories of higher externalizing problems among White youth. This is consistent with prior research showing that polygenic risk scores for adult externalizing were associated with externalizing behaviors among White adolescents and young adults (20), and that polygenic risk scores for childhood aggression were associated with trajectories of aggressive behaviors from childhood to early adulthood (22). We extend the literature by examining AdultExt-PRS and ChildAgg-PRS simultaneously and showing that they both uniquely predicted adolescent externalizing behaviors above and beyond the effect of each other. This suggests that there is developmental continuity in genetic influences on externalizing behaviors across development (57), whereby genetic risk for adult externalizing predicts externalizing behaviors earlier in development (i.e., from childhood to early adolescence). Further, these findings highlight that developmentally-matched polygenic scores have unique predictive value beyond polygenic scores derived from adult-based GWAS, suggesting developmentally-specific genetic influences. Interestingly, it also appears that AdultExt-PRS is more predictive of externalizing behavior than ChildAgg-PRS based on their effect sizes (odds ratios), although we did not statistically test the difference. There are more environmental influences on child phenotypes and most phenotypes (including externalizing behaviors) become more heritable with age as independence and agency increase over time. AdultExt-PRS may be able to capture more of the genetic influence on externalizing behaviors than ChildAgg-PRS because they capture variants of effect during adulthood. We note that the discovery GWAS for AdultExt-PRS was more powerful with a much larger sample size than the discovery GWAS for ChildAgg-PRS, which may also have contributed to their differential predictive ability. Overall, these findings suggest that incorporating adult-based and child-based polygenic scores is useful to capture genetic influences across development and improve polygenic prediction.

Polygenic effects were also found among Black and Hispanic youth. Specifically, AdultExt-PRS was significantly associated with greater likelihood of following trajectories of higher externalizing behaviors among Black youth, and ChildAgg-PRS was significantly associated with greater likelihood of following trajectories of higher externalizing behaviors among Hispanic youth. These findings are consistent with prior research showing that AdultExt-PRS predicted more externalizing behaviors among Black young adults and greater likelihood of developing any externalizing disorder among Mexican youth (21). Because polygenic scores were derived from GWAS with predominantly White participants and were not ancestrally aligned for Black and Hispanic youth, effects of polygenic scores tend to be weaker or non-significant among Black and Hispanic youth, as expected. One exception is that the effect of AdultExt-PRS appeared to be stronger in Black youth in predicting the likelihood of following the high externalizing behaviors trajectory. This may reflect G×E effects where Black youth might experience environments (e.g., institutional racism, structural racism) that exacerbate genetic influences. Future research is warranted to study the role of salient environmental factors (e.g., racism) in moderating genetic influences among Black youth. The lack of statistical significance for some of the effects of polygenic scores may also be due to smaller sample size and limited statistical power among the Black and Hispanic subgroups. For example, several PRS effects for Black and Hispanic youth have odds ratios of similar size with those statistically significant among White youth, but they were not statistically significant among Black and Hispanic youth likely due to smaller sample size and larger standard errors. We caution that it is important to evaluate the effect sizes of the coefficients instead of only focusing on statistical significance and *p* value.

Across racial-ethnic groups, family conflict was associated with higher risk of following trajectories of higher externalizing behaviors (Hypothesis 3). This finding is consistent with prior research showing the detrimental effect of family conflict on youth externalizing psychopathology across Black, White, and Hispanic youth (58). Also consistent with our hypothesis and prior research, parental acceptance was significantly associated with lower likelihoods of following trajectories of higher externalizing behaviors among White and Black youth. It is surprising that the associations between parental acceptance and trajectories of externalizing behaviors were not statistically significant among Hispanic youth, as prior research indicated parental acceptance and warmth play an important role among Hispanic youth. We note that the odds ratios are comparable with or larger than those observed for White and Black youth. The null significance as indicated by the p value greater than.05 could be due to smaller sample size and limited statistical power for the Hispanic subgroup. This might be particularly true given that the level of parental acceptance was relatively high on average (mean of 2.74 on a scale from 1 to 3) and variability was relatively small (standard deviation was.29). Contrary to our hypothesis, parental monitoring was generally not significantly associated with trajectories of externalizing behaviors across racial-ethnic groups, with one exception that higher parental monitoring was associated with lower likelihoods of following the high externalizing trajectory relative to the moderate externalizing trajectory among Black youth. Prior research indicated an important role of parental monitoring in adolescent externalizing behaviors among adolescents of diverse backgrounds (59, 60). However, most prior research did not simultaneously examine the role of multiple family processes, which may have overestimated the effect of parental monitoring. Perhaps some effect of parental monitoring may overlap with other aspects of family processes (i.e., shared variance among multiple family processes) or the effect of other family processes. We note that the internal consistency for the parental monitoring measure was low, indicating possible measurement errors, which may have contributed to the null finding.

In terms of gene-environment interplay, we did not find any significant G×E effects, suggesting that family processes did not moderate polygenic effects on trajectories of externalizing behaviors (Hypothesis 4). This finding is consistent with prior studies with the ABCD data where interactions effects between alcohol use disorder polygenic scores and family processes were also absent when predicting growth in externalizing behaviors (25). Notably, variations in family processes within the ABCD community sample are limited and largely within the normal range, with overall high levels of parental acceptance and monitoring and low levels of family conflict, which might have limited their potential to moderate genetic influences. Indeed, theories such as the diathesis-stress theory suggest that adverse environments such as stressful life events are more likely to moderate/exacerbate genetic risk. Future research can examine the role of extreme negative family processes such as parental neglect or maltreatment in moderating genetic effects, or examine the role of normative family practices in at-risk samples. Research is also needed to examine the role of environmental factors beyond the family environment in moderating genetic effects, such as peer factors, neighborhood context, and experiences of institutional and structural racism.

Findings revealed some evidence of rGE among White youth (Hypothesis 5). Both AdultExt-PRS and ChildAgg-PRS were associated with higher family conflict, which in turn were associated with higher likelihood of following the high increasing externalizing trajectory among White youth. These findings are consistent with prior research showing that White adolescents’ PRS for externalizing problems predicted greater family dysfunction which in turn predicted more externalizing problems in young adulthood (27). These findings may reflect evocative rGE where adolescents’ genetic predispositions evoke responses from their family environment. However, because parents’ genetic predispositions were not examined in the present study, we were not able to tease apart passive vs. evocative rGE. Nonetheless, these findings add to the growing literature demonstrating rGE as important mechanisms underlying the development of externalizing psychopathology (7). No significant rGE was found among Black and Hispanic youth, which could be due to diminished predictibility of PRS among these groups. This further highlights the importance of conducting genetically-informed research in diverse populations, including the need to improve diversity in GWAS studies in order to better characterize polygenic risk scores among diverse populations.

This study extends the literature by taking a developmental approach to examine the role of both adult-based and child-based polygenic scores in predicting different trajectories of externalizing behaviors from late childhood to early adolescence. Notable strengths of the study include the simultaneous consideration of multiple aspects of family processes and studying different forms of gene-environment interplay (i.e., G×E and rGE) among racially and ethnically diverse youth. Findings need to be interpreted in light of several limitations. First, polygenic scores were derived based on GWAS conducted among individuals of European ancestry. Given evidence that polygenic scores based on GWAS of European ancestry samples can be biased and have reduced predictive power when applied to non-European ancestry samples, our findings on polygenic effects among Black and Hispanic youth should be interpreted with caution. In particular, weaker or non-significant polygenic effects observed among these subgroups do not indicate that genetic influences are less or not important for Black or Hispanic youth; rather, they likely reflect methodological limitations and highlight the need for increased representation of Black and Hispanic populations in GWAS studies and genetically informed research more broadly. Future GWAS studies on externalizing behaviors with trans-ancestry samples are warranted, and research is needed to apply methods such as PRS-CSx (61) and TL-PRS (62) to improve accuracy and prediction of PRS across populations. Second, although the ABCD sample is national and large, it is a community sample with youth generally exhibiting relatively low levels of externalizing behaviors, low family conflict, and high parental acceptance and monitoring. Findings from the present study may not generalize to youth and families at high-risk or in clinical settings. Third, the measures of family processes, parental monitoring in particular, have lower internal consistency and reliability. Measurement error could have contributed to these lower reliabilities and may have resulted in bias in the findings. Fourth, we only used four waves of data capturing externalizing behaviors from age 9/10 to 12/13. Research with additional waves of longitudinal data would be useful to understand gene-environment interplay underlying trajectories of externalizing behaviors from childhood through adolescence and adulthood. In addition, only family processes at the baseline assessment were examined. Future research should include family processes across multiple waves to examine the reciprocal associations between family processes and externalizing behaviors over time. Finally, although GMM is a popular approach to capture heterogeneity in developmental trajectories, we recognize that this approach has been criticized for potentially generating artifactual groups (63). Future research is needed to replicate the current findings with GMM and other longitudinal analytic approaches.

### 4.1 Conclusion

There is substantial heterogeneity in trajectories of externalizing behaviors from late childhood to early adolescence. Our findings indicated that both adult-based and child-based polygenic scores for externalizing behaviors are useful in predicting trajectories of externalizing behaviors, highlighting developmental continuity in genetic influences. Family processes, especially family conflict, play an important role in adolescent externalizing behaviors across racial/ethnic groups, suggesting the need to target family conflict in intervention efforts.

## Data Availability

Publicly available datasets were analyzed in this study. This data can be found here: The ABCD data used in this report came from NIMH Data Archive Release 5.1(http://dx.doi.org/10.15154/z563-zd24).
