# Supplementary material for: Interplay between polygenic risk and family processes in predicting trajectories of adolescent externalizing behaviors
Source: Front Psychiatry. 2025 Mar 12;16:1505035. doi: 10.3389/fpsyt.2025.1505035 (PMC11937852; doi:10.3389/fpsyt.2025.1505035)
Supplement: Supplementary file 1 [file Table1.docx]

Supplemental Table 1

Prevalence and Estimated Intercept and Linear Slope Across Trajectories of Adolescent Externalizing Behaviors

|  |  | **Intercept** | | **Linear Slope** | |
| --- | --- | --- | --- | --- | --- |
|  | N (%) | Estimate | *p* value | Estimate | *p* value |
| **White Youth** |  |  |  |  |  |
| High Increasing | 482 (8.2%) | 61.01 | < .001 | .82 | .014 |
| Moderate | 2139 (36.2%) | 49.13 | < .001 | .34 | .034 |
| Low Decreasing | 3284 (55.6%) | 40.45 | < .001 | -.88 | <.001 |
| **Black Youth** |  |  |  |  |  |
| High | 166 (9.8%) | 63.90 | < .001 | -.01 | .980 |
| Moderate | 556 (32.8%) | 51.61 | < .001 | -.31 | .386 |
| Low Decreasing | 971 (57.4%) | 41.16 | < .001 | -1.41 | < .001 |
| **Hispanic Youth** |  |  |  |  |  |
| High | 120 (5.7%) | 60.31 | < .001 | 1.45 | .064 |
| Moderate | 781 (36.9) | 50.55 | < .001 | .22 | .260 |
| Low Decreasing | 1215 (57.4%) | 41.53 | < .001 | -1.19 | < .001 |

Supplemental Table 2

Coefficients from Path Models Predicting Membership in Trajectories of Externalizing Behaviors from Polygenic Scores and Family Processes among White Youth

| **Paths** | **Beta** | **95% CI** | ***p*** |
| --- | --- | --- | --- |
| AdultExt-PRS 🡪 High Increasing Trajectory | .14 | .10, .18 | < .001 |
| ChildAgg-PRS 🡪 High Increasing Trajectory | .08 | .02, .13 | .006 |
| AdultExt-PRS 🡪 Moderate Trajectory | .08 | .06, .11 | < .001 |
| ChildAgg-PRS 🡪 Moderate Trajectory | .06 | .03, .08 | < .001 |
| Family Conflict 🡪 High Increasing Trajectory | .15 | .08, .21 | < .001 |
| Parental Acceptance 🡪 High Increasing Trajectory | -.06 | -.11, -.10 | .019 |
| Parental Monitoring 🡪 High Increasing Trajectory | -.04 | -.09, .01 | .081 |
| Family Conflict 🡪 Moderate Trajectory | .05 | .03, .08 | < .001 |
| Parental Acceptance 🡪 Moderate Trajectory | -.04 | -.07, -.01 | .023 |
| Parental Monitoring 🡪 Moderate Trajectory | -.00 | -.05, .04 | .922 |
| **AdultExt-PRS 🡪 Family Conflict** | **.03** | **.01, .06** | **.015** |
| **ChildAgg-PRS 🡪 Family Conflict** | **.03** | **.00, .06** | **.041** |
| **AdultExt-PRS 🡪 Parental Acceptance** | **-.04** | **-.06, -.01** | **.008** |
| ChildAgg-PRS 🡪 Parental Acceptance | -.02 | -.05, .01 | .195 |
| AdultExt-PRS 🡪 Parental Monitoring | -.02 | -.04, .01 | .274 |
| ChildAgg-PRS 🡪 Parental Monitoring | .00 | -.03, .03 | 1.00 |
| **Indirect Effects** |  |  |  |
| AdultExt-PRS 🡪 Family Conflict 🡪 High Increasing Trajectory | .005 | .000, .010 | .062 |
| AdultExt-PRS 🡪 Parental Acceptance 🡪 High Increasing Trajectory | .002 | .000, .004 | .082 |
| AdultExt-PRS 🡪 Parental Monitoring 🡪 High Increasing Trajectory | .001 | -.001, .002 | .375 |
| ChildAgg-PRS 🡪 Family Conflict 🡪 High Increasing Trajectory | **.004** | **.001, .008** | **.025** |
| ChildAgg-PRS 🡪 Parental Acceptance 🡪 High Increasing Trajectory | .001 | -.001, .003 | .298 |
| ChildAgg-PRS 🡪 Parental Monitoring 🡪 High Increasing Trajectory | .000 | -.001, .001 | 1.00 |
| AdultExt-PRS 🡪 Family Conflict 🡪 Moderate Trajectory | .002 | .000, .004 | .037 |
| AdultExt-PRS 🡪 Parental Acceptance 🡪 Moderate Trajectory | .001 | .000, .003 | .082 |
| AdultExt-PRS 🡪 Parental Monitoring 🡪 Moderate Trajectory | .000 | -.001, .001 | .925 |
| ChildAgg-PRS 🡪 Family Conflict 🡪 Moderate Trajectory | .002 | .000, .003 | .085 |
| ChildAgg-PRS 🡪 Parental Acceptance 🡪 Moderate Trajectory | .001 | -.001, .002 | .286 |
| ChildAgg-PRS 🡪 Parental Monitoring 🡪 Moderate Trajectory | .000 | .000, .000 | 1.00 |

Note. The low decreasing trajectory was the reference group in the path model. Significant indirect effects (based on 95% CI) reflecting rGE in predicting externalizing trajectories are bolded.

Supplemental Table 3

Coefficients from Path Models Predicting Membership in Trajectories of Externalizing Behaviors from Polygenic Scores and Family Processes among Black Youth

| **Paths** | **Beta** | **95% CI** | ***p*** |
| --- | --- | --- | --- |
| AdultExt-PRS 🡪 High Trajectory | .15 | .09, .22 | < .001 |
| ChildAgg-PRS 🡪 High Trajectory | .07 | .01, .14 | .028 |
| AdultExt-PRS 🡪 Moderate Trajectory | .04 | -.04, .11 | .310 |
| ChildAgg-PRS 🡪 Moderate Trajectory | .01 | -.04, .05 | .733 |
| Family Conflict 🡪 High Trajectory | .11 | .03, .19 | .007 |
| Parental Acceptance 🡪 High Trajectory | -.01 | -.07, .05 | .818 |
| Parental Monitoring 🡪 High Trajectory | -.06 | -.12, -.01 | .031 |
| Family Conflict 🡪 Moderate Trajectory | .01 | -.05, .06 | .754 |
| Parental Acceptance 🡪 Moderate Trajectory | -.08 | -.14, -.02 | .011 |
| Parental Monitoring 🡪 Moderate Trajectory | .05 | -.05, .15 | .362 |
| AdultExt-PRS 🡪 Family Conflict | .00 | -.05, .06 | .981 |
| ChildAgg-PRS 🡪 Family Conflict | .01 | -.02, .03 | .677 |
| AdultExt-PRS 🡪 Parental Acceptance | -.00 | -.05, .04 | .856 |
| ChildAgg-PRS 🡪 Parental Acceptance | .00 | -.05, .05 | .973 |
| AdultExt-PRS 🡪 Parental Monitoring | -.00 | -.03, .03 | .972 |
| ChildAgg-PRS 🡪 Parental Monitoring | -.01 | -.04, .03 | .685 |
| **Indirect Effects** |  |  |  |
| AdultExt-PRS 🡪 Family Conflict 🡪 High Trajectory | .000 | -.006, .006 | .981 |
| AdultExt-PRS 🡪 Parental Acceptance 🡪 High Trajectory | .000 | .000, .000 | .901 |
| AdultExt-PRS 🡪 Parental Monitoring 🡪 High Trajectory | .000 | -.002, .002 | .972 |
| ChildAgg-PRS 🡪 Family Conflict 🡪 High Trajectory | .001 | -.003, .004 | .704 |
| ChildAgg-PRS 🡪 Parental Acceptance 🡪 High Trajectory | .000 | .000, .000 | .973 |
| ChildAgg-PRS 🡪 Parental Monitoring 🡪 High Trajectory | .000 | -.002, .003 | .695 |
| AdultExt-PRS 🡪 Family Conflict 🡪 Moderate Trajectory | .000 | .000, .000 | .981 |
| AdultExt-PRS 🡪 Parental Acceptance 🡪 Moderate Trajectory | .000 | -.003, .004 | .857 |
| AdultExt-PRS 🡪 Parental Monitoring 🡪 Moderate Trajectory | .000 | -.001, .001 | .971 |
| ChildAgg-PRS 🡪 Family Conflict 🡪 Moderate Trajectory | .000 | .000, .000 | .810 |
| ChildAgg-PRS 🡪 Parental Acceptance 🡪 Moderate Trajectory | .000 | -.004, .004 | .973 |
| ChildAgg-PRS 🡪 Parental Monitoring 🡪 Moderate Trajectory | .000 | -.002, .001 | .666 |

Note. The low decreasing trajectory was the reference group in the path model.

Supplemental Table 4

Coefficients from Path Models Predicting Membership in Trajectories of Externalizing Behaviors from Polygenic Scores and Family Processes among Hispanic Youth

| **Paths** | **Beta** | **95% CI** | ***p*** |
| --- | --- | --- | --- |
| AdultExt-PRS 🡪 High Trajectory | .08 | -.00, .16 | .060 |
| ChildAgg-PRS 🡪 High Trajectory | .09 | -.02, .20 | .117 |
| AdultExt-PRS 🡪 Moderate Trajectory | .02 | -.03, .06 | .402 |
| ChildAgg-PRS 🡪 Moderate Trajectory | .07 | .01, .13 | .022 |
| Family Conflict 🡪 High Trajectory | -.03 | -.14, .08 | .539 |
| Parental Acceptance 🡪 High Trajectory | -.07 | -.16, .02 | .132 |
| Parental Monitoring 🡪 High Trajectory | -.03 | -.11, .06 | .537 |
| Family Conflict 🡪 Moderate Trajectory | .08 | .02, .13 | .010 |
| Parental Acceptance 🡪 Moderate Trajectory | -.04 | -.08, -.00 | .039 |
| Parental Monitoring 🡪 Moderate Trajectory | .02 | -.07, .11 | .654 |
| AdultExt-PRS 🡪 Family Conflict | .02 | -.01, .04 | .287 |
| ChildAgg-PRS 🡪 Family Conflict | .00 | -.05, .06 | .864 |
| AdultExt-PRS 🡪 Parental Acceptance | .02 | -.02, .07 | .354 |
| ChildAgg-PRS 🡪 Parental Acceptance | -.01 | -.05, .03 | .758 |
| AdultExt-PRS 🡪 Parental Monitoring | .02 | -.02, .07 | .264 |
| ChildAgg-PRS 🡪 Parental Monitoring | .01 | -.02, .05 | .479 |
| **Indirect Effects** |  |  |  |
| AdultExt-PRS 🡪 Family Conflict 🡪 High Trajectory | -.001 | -.002, .001 | .568 |
| AdultExt-PRS 🡪 Parental Acceptance 🡪 High Trajectory | -.002 | -.005, .002 | .391 |
| AdultExt-PRS 🡪 Parental Monitoring 🡪 High Trajectory | -.001 | -.002, .001 | .509 |
| ChildAgg-PRS 🡪 Family Conflict 🡪 High Trajectory | .000 | -.002, .002 | .862 |
| ChildAgg-PRS 🡪 Parental Acceptance 🡪 High Trajectory | .000 | -.002, .003 | .743 |
| ChildAgg-PRS 🡪 Parental Monitoring 🡪 High Trajectory | .000 | -.001, .001 | .573 |
| AdultExt-PRS 🡪 Family Conflict 🡪 Moderate Trajectory | .001 | -.001, .003 | .304 |
| AdultExt-PRS 🡪 Parental Acceptance 🡪 Moderate Trajectory | -.001 | -.003, .001 | .412 |
| AdultExt-PRS 🡪 Parental Monitoring 🡪 Moderate Trajectory | .001 | -.001, .003 | .680 |
| ChildAgg-PRS 🡪 Family Conflict 🡪 Moderate Trajectory | .000 | -.003, .004 | .862 |
| ChildAgg-PRS 🡪 Parental Acceptance 🡪 Moderate Trajectory | .000 | -.001, .002 | .765 |
| ChildAgg-PRS 🡪 Parental Monitoring 🡪 Moderate Trajectory | .000 | -.001, .001 | .618 |

Note. The low decreasing trajectory was the reference group in the path model.
